# Risk of Fatal Bleeding in Episodes of Major Bleeding with New Oral Anticoagulants and Vitamin K Antagonists: A Systematic Review and Meta-Analysis

**DOI:** 10.1371/journal.pone.0137444

**Published:** 2015-09-18

**Authors:** Joel Skaistis, Travis Tagami

**Affiliations:** Department of Internal Medicine, William Beaumont Hospital, Royal Oak, Michigan, United States of America; University of Perugia, ITALY

## Abstract

**Background:**

The reversibility of new/novel oral anticoagulants (NOAC) is not well understood, whereas the reversal strategies for bleeding associated with vitamin k antagonists (VKA), such as warfarin, is well established. It is unknown whether outcomes are different between bleeds occurring with NOAC compared to VKA use.

**Objectives:**

This systematic review and meta-analysis of randomized controlled trials determines the relative odds of fatal bleeding given that a patient suffered a major bleed while on NOAC versus VKA therapy.

**Search Methods:**

Data on major and fatal bleeding events was sought from randomized controlled trials of NOAC agents compared to VKAs.

**Main Results:**

20 trials were included in the meta-analysis. From which, 4056 first-time, major bleeding events were reported and included in the primary analysis. The summary odds ratio for the conditional odds of fatal bleeding given that a major bleeding event occurred was 0.65 [0.52, 0.81] favoring the NOAC agents (p = 0.0001). The reduced odds of fatal bleeding with NOACs was not demonstrated after controlling for bleeding location. Given that an intracranial bleeding event occurred, the summary odds ratio for the conditional odds of fatal bleeding was 0.96 [0.70, 1.32]. For extracranial bleeding events, the summary odds ratio was also statistically insignificant at 0.945 [0.66, 1.35].

**Author’s Conclusions:**

The odds ratio calculated in this meta-analysis showed a reduced odds of death in major bleeding associated with NOAC use. This risk reduction was due to a disproportionate amount of intracranial bleeding in the VKA arms. For any given bleeding site, there was no evidence of a significant difference in fatal outcomes from bleeds associated with NOAC versus VKA use.

**Protocol Registration:**

Protocol registered on PROSPERO under CRD42014013294.

## Introduction

Several new oral anticoagulants (NOAC) have been approved and adopted into routine clinical practice since the approval of dabigatran by the Food and Drug Administration in 2010. These medications have been approved as alternatives to vitamin K antagonists (VKA) such as warfarin for prevention of stroke and systemic embolism in atrial fibrillation and for treatment of venous thromboembolism. Four medications have currently been studied in phase III trials, apixaban, dabigatran, edoxaban and rivaroxaban. Apixaban, edoxaban and dabigatran have shown superior efficacy compared to VKAs for prevention of stroke and embolism and rivaroxaban has shown comparable efficacy [1–4]. As with VKAs, bleeding is the main adverse outcome seen with the NOACs. Lower bleeding rates compared to VKAs were seen with apixaban and edoxaban and comparable bleeding rates were seen with dabigatran and rivaroxaban. Major bleeding rates with these medications ranged from 1% to 3.6% per year [4,5].

A frequent concern with prescribing any of the new oral anticoagulants (NOACs) is that there is no well-established antidote, whereas the reversal of VKAs is well understood and detailed in professional society guidelines [6]. Coagulation markers are less than optimal for guiding attempts at NOAC reversal. Normalization of these markers does not consistently reflect neutralization of the anticoagulation effects of the NOACs. Trials of prothrombin complex concentrate (PCC) demonstrated reversal of aPTT within 15 minutes of infusion in healthy volunteers taking rivaroxaban [7,8]. This same trial showed that for volunteers treated with dabigatran, PCC failed to reverse changes in the aPTT, thrombin time and ecarin clotting time. Despite this lack of any coagulation marker reversal, animal studies have shown PCC to be effective at achieving clinical hemostasis in dabigatran-bleeding models [9]. Activated prothrombin complex concentrate (aPCC or FEIBA) and recombinant factor VIIa (rFVIIa) also showed achievement of hemostasis without reversal of coagulation markers in a dabigatran-treated animal bleed model [10]. In vitro assays of volunteers treated with apixaban showed improvements with administration of PCC, aPCC and rFVIIa [11]. However, in an animal bleed model done with apixaban, rFVIIa and PCC reversed changes in coagulation markers but failed to improve hemostasis [12]. A recombinant protein r-Antidote, has also been developed for anticoagulation reversal in NOAC treated patients. This protein is a catalytically inactive form of the factor Xa receptor and has been shown to reverse coagulation markers in human plasma and maintain hemostasis in rivaroxaban treated rats [13].

Some limited data regarding bleeding risk with commercial NOAC use is available. The Institute for Safe Medication Practices regularly reviews the FDA MedWatch adverse reaction reporting database. Among the NOACs, they have identified alarming reports regarding dabigatran use. However, these reports are highly biased due to the voluntary nature of the reports from which they are generated. In the first quarter of 2011, soon after its approval date, dabigatran accounted for 505 reported cases of severe bleeding, which was the highest number of adverse events for all regularly monitored drugs in that quarter. Warfarin had the second highest number of adverse event reports at 176 [14]. A 2011 study showed that 542 of 2367 (23%) reported hemorrhagic cases on dabigatran were fatal while only 72 of 731 (10%) reported hemorrhagic cases on a VKA were fatal [15]. The novelty and increased publicity surrounding the release of dabigatran to the market would be expected to result in similarly increased vigilance for adverse events related to its use.

This systematic review and meta-analysis calculates the relative odds of fatal bleeding after occurrence of major bleeding during NOAC or VKA treatment. It includes studies of patients receiving anticoagulation for thromboembolism prophylaxis due to history of atrial fibrillation or venous thromboembolism. Apixaban, dabigatran, edoxaban and rivaroxaban were compared to VKAs to determine the aggregate effect size for the ratio of odds of fatal bleeding in patients with major, intracranial and major extracranial bleeding. Aggregate patient outcome data was collected from randomized controlled trials for meta-analysis.

## Methods

The protocol for this review is registered on PROSPERO under CRD42014013294 [16]. Inclusion criteria are given in Table 1.

**Table 1:** Study inclusion criteria.

Study type	Randomized controlled trials
Participants	Age>18; Patient with atrial fibrillation or acute venous thromboembolism; Anticoagulation indicated for either prophylaxis of stroke and systematic thromboembolism in atrial fibrillation or treatment of acute venous thromboembolism
Interventions	Oral anticoagulation with any of the following NOAC agents: dabigatran, rivaroxaban, apixaban or edoxaban
Control	VKA
Outcomes	Number of patients with major bleeding events as defined by the International Society on Thrombosis and Hemostasis (ISTH) criteria (clinically overt bleeding with a decrease in hemoglobin of >2 g/dL in a 24 hour period; transfusion of 2 or more units of packed red blood cells; bleeding that occurred in any of the following critical sites: intracranial, intraspinal, intraocular, pericardial, intra-articular, intramuscular with compartment syndrome and retroperitoneal; fatal bleeding); Number of patients with major intracranial bleeding events; Number of patients with major intracranial bleeding events; Number of fatal intracranial bleeding events; Number of fatal extracranial bleeding events

A single reviewer searched the following databases: Pubmed, Cochrane (CENTRAL), Ovidweb, Clinicaltrial.gov register, ISRCTN register, World Health Organization ICTRP register. The databases were searched with the following MeSH terms, keywords and limits: pulmonary embolism, atrial fibrillation, stroke, venous thromboembolism, embolism, embolism and thrombosis, dabigatran, edoxaban, rivaroxaban, apixaban, Randomized control trial. Searches were carried out on 8/20/2014 and 8/21/2014.

Only English language studies were included. No publication date or publication restrictions were imposed. Trials without major bleeding events in either treatment arm were excluded from the primary analysis.

## Data Collection and Analysis

Two reviewers extracted data independently according to a prespecified data extraction sheet (S1 Table). Authors of studies were contacted to provide missing data. Events occurring in treatment arms assigned to drug dosages that failed to gain FDA approval were excluded from the analysis. Analyses were performed using the number of patients with major bleeding events as well as total number of major events. For studies that did not have data on the total major events available, this value was approximated by the sum of the number of patients with bleeding at individual bleeding locations. For studies that did not analyze extracranial bleeding outcomes, estimated extracranial bleeding was calculated from the number of patients with major bleeding and no intracranial bleeding.

Study validity was assessed with the Cochrane Collaboration’s risk of bias assessment tool [17]. The following domains were considered: sequence generation; allocation concealment; blinding of participants, personnel and outcome assessors; incomplete outcome data. Validity was assessed at the study level. Validity was not assessed for individual outcomes.

Data extracted from trials was combined by a random effects model with effect sizes given by the ratio of the odds of a fatal bleed in the NOAC treatment arm given occurrence of a major bleed relative to the odds of a fatal bleed in the control arm given occurrence of a major bleed. Total effect size was calculated by the Mantel-Haenszel method. Heterogeneity was evaluated with I^2^ calculations. Statistical analysis was performed with RevMan 5.3 (Copenhagen: The Nordic Cochrane Centre, The Cochrane Collaboration, 2014.) with two tailed p-values <0.05 considered significant. Drug type, anticoagulation indication and degree of blinding were used in subgroup analysis. For studies with multiple treatment arms, analysis was performed with the events of multiple treatment arms pooled together. Sensitivity analysis was performed with events from each treatment arm treated as separate, independent studies and the events in the control arm split in proportion to the populations of the treatment arms. In the primary analysis, total major bleeding events were approximated by the number of patients with major bleeds. Sensitivity analysis was performed with the number of total major bleeding events or with the sum of patients with major bleeds at given anatomic locations if the absolute number of total bleeding events was not available. Studies with zero fatal bleeds in both study arms were excluded from the primary analysis. Sensitivity analysis was performed with substitution of 0.5 for the number of fatal bleeds. Studies without major bleeding events in both trial arms were excluded from the primary analysis. Sensitivity analysis was performed with the events from these studies pooled into a single trial.

No publication bias assessment was performed. A comprehensive search of trial registries was performed to ensure both published and unpublished data was included. Study authors were contacted for additional results to correct for reporting bias.

## Results

### Results of the Search

**Fig 1:**
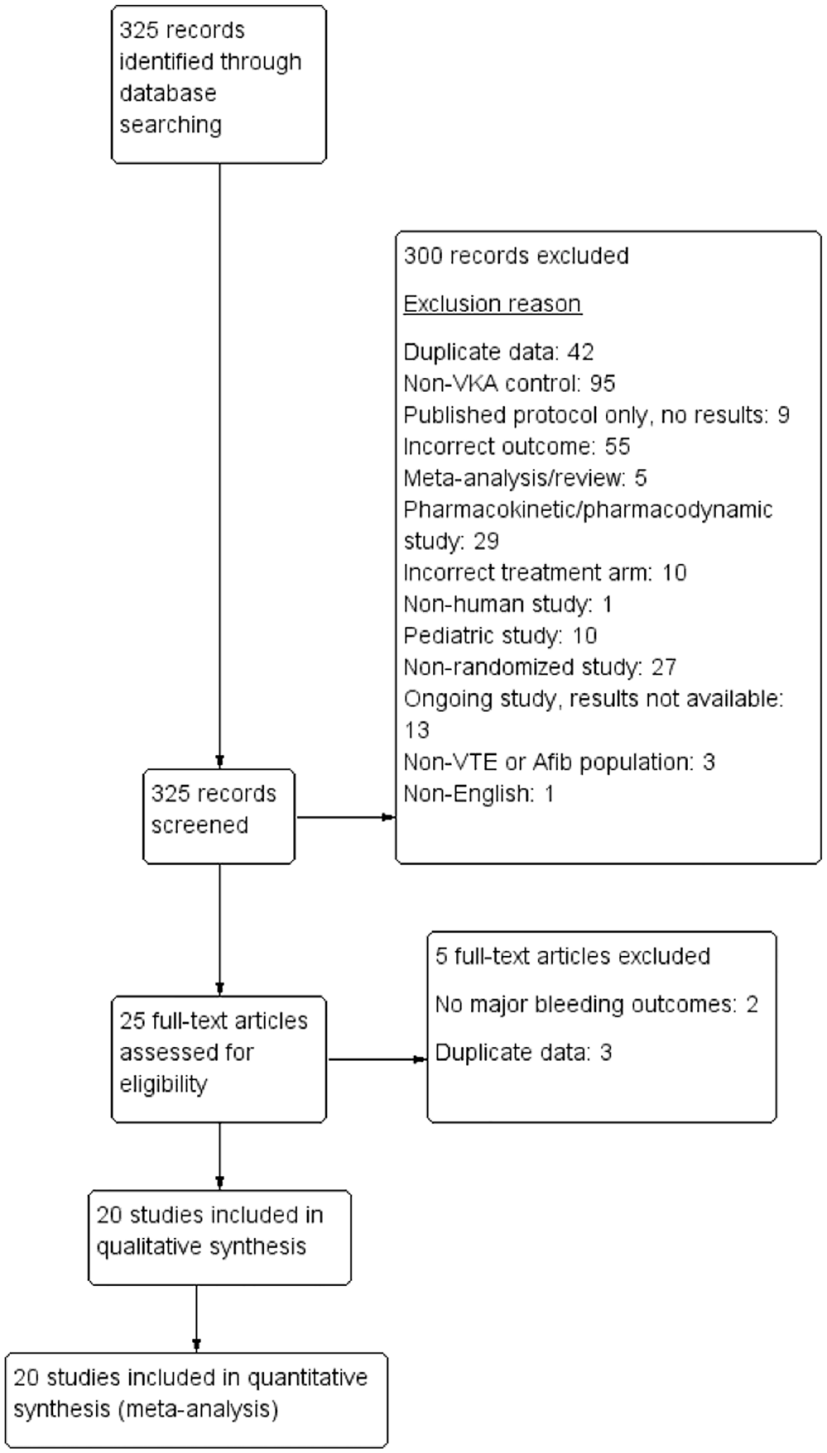
Study selection flowsheet. Flowsheet of the study selection process according to PRISMA guidelines.

325 records were identified from the literature search (Fig 1). 300 records were excluded during the abstract review phase. 25 full text studies were reviewed. 5 of these studies were excluded and 20 studies met inclusion criteria [2–6,18–35].

### Risk of Bias in Included Studies

Studies overall were of high methodological quality. All were adequately blinded. The J-ROCKET AF trial lacked adequate description of the sequence generation and allocation process. All other studies were at low risk for bias in this criteria. Outcome data was complete in all studies except the RE-LY trial and NCT01136408, which were rated as high risk for bias due to significantly more early withdrawals in the NOAC arm. Characteristics and risk of bias of studies is detailed in S1, S2 and S3 Figs.

### Effects of Interventions

**Fig 2:**
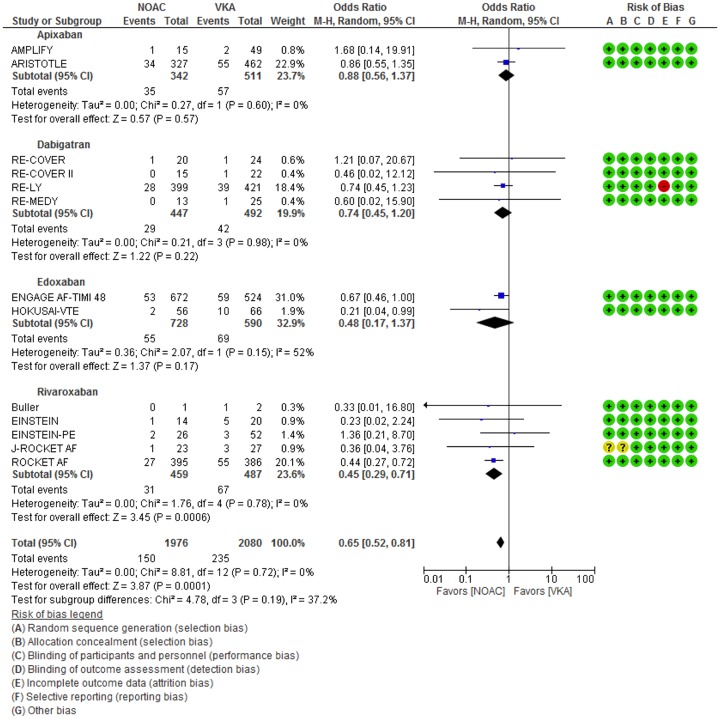
Relative Odds of Fatal Bleeding Given Occurrence of Major Bleed. Odds ratios and 95% confidence intervals are given for the relative odds that major bleeding events will lead to fatal bleeding events.

Baseline characteristics of the included studies are given in Table 2. 4056 major bleeding events were identified from 13 studies and were included in the primary meta-analysis. The total number of first time major bleeding events between NOAC and VKA arms was similar with 1976 events in the NOAC arms and 2080 events in the VKA arms. 150 NOAC-associated, fatal bleeding events occurred versus 235 VKA-associated events. Across all included studies, the odds ratio for fatal bleeding given that a patient had experienced a major bleed ranged from 0.21 in favor of NOACs to 1.68 in favor of VKAs (Fig 2). The summary odds ratio was 0.65 [0.52, 0.81] favoring NOACs, which was statistically significant with p = 0.0001. Across the individual medication subgroups, only rivaroxaban achieved a statistically significant decreased odds of death in patients with major bleeding compared to VKA control. All other medication subgroups showed decreased odds for fatal bleeding with NOAC agents but these differences were not statistically significant. The effect sizes were homogeneous across the studies with an I^2^ value of 0%. Homogeneity was retained between medication subgroups. I^2^ value was 37.2% for subgroup heterogeneity.

**Table 2:** Baseline characteristics of included studies. Percentages represent the proportion of patients in each study with the given characteristic. Values are given for all study participants, not exclusively those who had major bleeds.

Study ID	Average age (years)	% Age > 75	% GFR<50 mL/min	% Previous CVA/TIA	% Concomitant antiplatelet use	Anticoagulation indication
ARISTOTLE	70	31.2%	16.5%	19.2%	NR	AFIB
ENGAGE AF-TIMI 48	72	40.5%	19.6%	28.1%	29.4%	AFIB
J-ROCKET AF	71	39.4%	22.1%	63.8%	38.0%	AFIB
RE-LY	71.5	40.1%	19.4%	20.3%	19.6%	AFIB
ROCKET AF	73	43.5%	21.0%	54.9%	34.9%	AFIB
ARISTOTLE-J	70.9	31.1%	NR	31.6%	26.8%	AFIB
Chung	65.2	12.3%	19.4%	14.3%	38.0%	AFIB
Weitz	65.5	NR	NR	NR	0.52	AFIB
Yamashita	68.6	0.28	0.12	0.3	0.26	AFIB
NCT01136408	67.85	NR	NR	NR	NR	AFIB
Buller	57.5	NR	NR	NR	NR	DVT
EINSTEIN	55.8	12.4%	7.0%	NR	NR	DVT
Agnelli	58.5	NR	NR	NR	0.0%	DVT
BOTICELLI	57.5	NR	NR	NR	NR	DVT
EINSTEIN-PE	57.9	18.2%	8.7%	NR	NR	PE
AMPLIFY	57.2	14.9%	6.5%	NR	NR	VTE
HOKUSAI-VTE	55.7	13.6%	6.5%	NR	8.9%	VTE
RE-COVER	55	9.9%	5.1%	NR	10.0%	VTE
RE-COVER II	54.7	9.9%	5.1%	NR	10.2%	VTE
RE-MEDY	55.4	9.8%	4.1%	2.2%	NR	VTE

NR = Not reported

**Fig 3:**
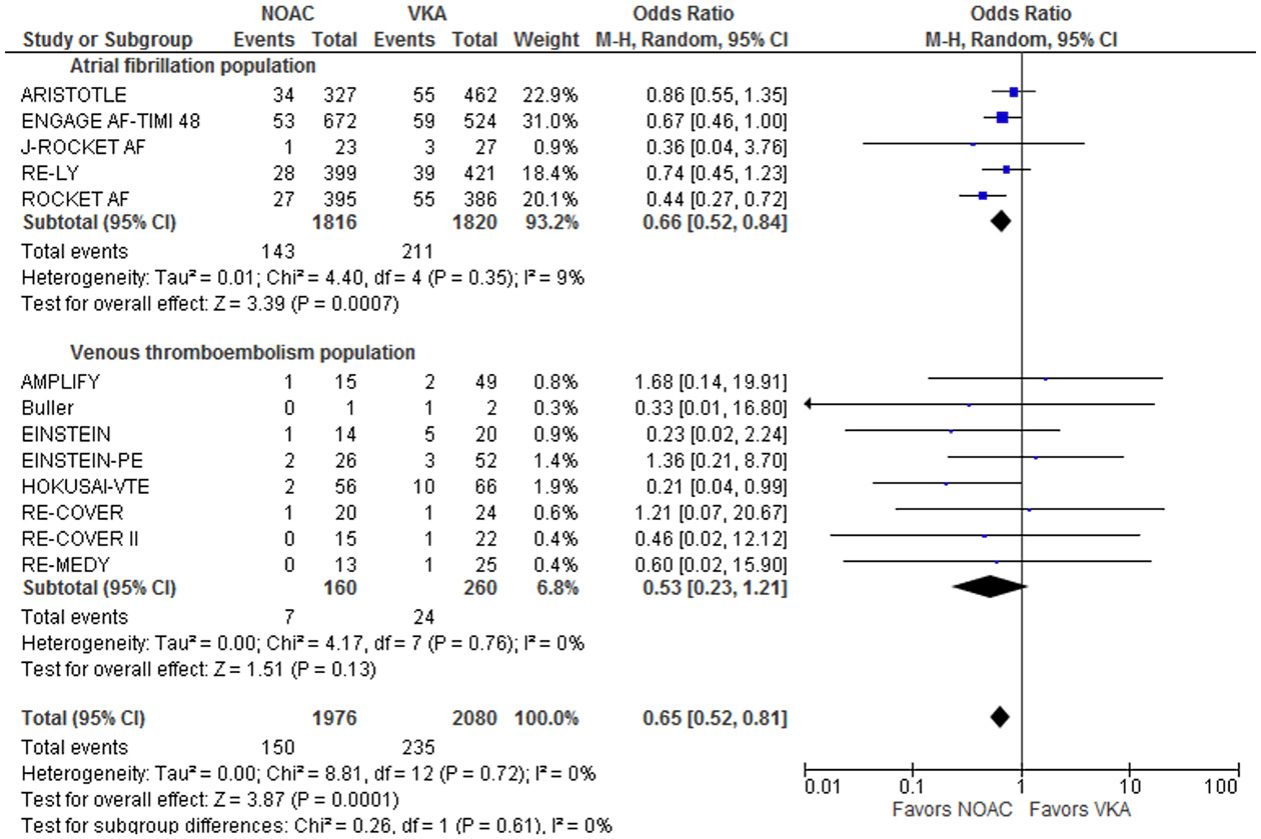
Relative Odds for fatal bleeding in treatment indication subgroups. Odds ratios and 95% confidence intervals are given for the relative odds that major bleeding events will lead to fatal bleeding events. Subgroups are determined by the indication for anticoagulation and fall into two categories, treatment of acute venous thromboembolism or stroke prophylaxis in atrial fibrillation.

When controlling for the indication for anticoagulation, odds ratios were similar in both atrial fibrillation and venous thromboembolism populations. The summary ratio for conditional odds of death was 0.66 [0.52, 0.84] (p = 0.0007) in all trials of atrial fibrillation populations and was similar for trials of venous thromboembolism populations at 0.53 [0.23, 1.21] (p = 0.13). (Fig 3). There was no heterogeneity between these subgroups and the I^2^ value for subgroup differences was 0%. Age was significantly different between these two groups. Average age in the atrial fibrillation population was 71.5 years and 56.1 years in the venous thromboembolism population (p<0.0001). Subgroups of the three open-label studies compared to the 10 double-blinded studies also showed no heterogeneity with I^2^ of 0%. (S4 Fig).

Alternative methods of calculating the odds ratio for fatal bleeding given the occurrence of major bleeding were explored in sensitivity analyses. In the primary analysis (Fig 2), the total number of major bleeding episodes was approximated by the number of patients with major bleeding episodes. Data was available from the RE-LY trial on the total number of major bleeding events. In addition, an alternative and closer approximation of total bleeds was calculated from the sum of first major bleeds at given anatomic locations for eight of the included studies. Substitution of total events for patients with major events yielded a total odds ratio of 0.65 [0.52, 0.80] (p<0.0001) (S5 Fig). This odds ratio is changed minimally from the primary analysis. The NCT01136408 study was excluded from the primary analysis because neither arm had a fatal event. Addition of this study to the meta-analysis minimally decreased the odds ratio to 0.65 [0.53, 0.81] (p<0.0001). Five studies had major bleeding episodes in only one study arm and individual odds ratios could not be calculated. After pooling these studies and including them in the meta-analysis, the total odds ratio was unchanged at 0.65 [0.52, 0.81] (p<0.0001).

In the primary analysis, the ENGAGE AF TIMI 48 study had two treatment arms that were combined into a single pair-wise comparison with the VKA control. The analysis was repeated with the two arms treated as separate trials and the shared VKA control split in proportion to the size of each treatment arm. The edoxaban subgroup odds ratio decreased from 0.48 [0.17, 1.37] (p = 0.17) to 0.63 [0.42, 0.94] (p = 0.02) while the total odds ratio was unchanged at 0.65 [0.52, 0.81] (p = 0.0001) (S6 Fig).

**Fig 4:**
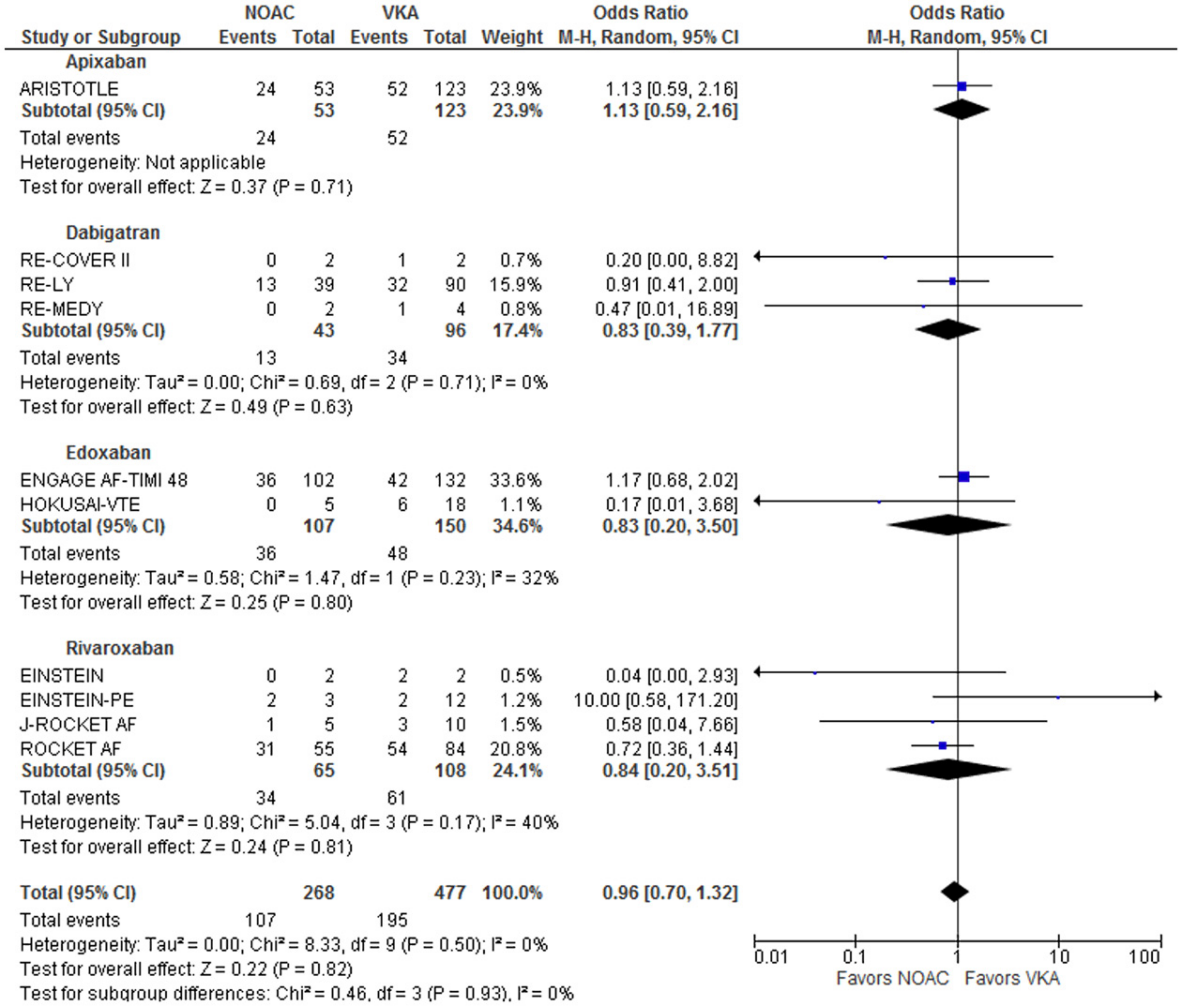
Relative odds of fatal bleeding given patient with intracranial bleeding event. Odds ratios and 95% confidence intervals are given for the relative odds that intracranial bleeding events will lead to fatal bleeding events.

Across the 10 studies that observed at least one intracranial bleed per treatment arm, there were 745 participants with intracranial bleeding events. 302 of these events resulted in death. The total odds ratio for fatal bleeding given an intracranial bleed occurred was 0.96 [0.70, 1.32] (p = 0.96) (Fig 4). No clinical or statistically significant difference was detected between the odds of fatal bleeding on NOAC and VKA agents. Testing for heterogeneity resulted in an I^2^ value of 0%.

Sensitivity analysis was performed for intracranial bleeding. Five studies had intracranial bleeding events in only one study arm and were excluded from the primary analysis. After adding the pooled results of these studies to the meta-analysis, the odds ratio increased minimally to 0.98 [0.71, 1.34] (p = 0.88). Adjustment for multiple treatment arms resulted in no change in the total odds ratio which remained at 0.96 [0.70, 1.32] (p = 0.79). The edoxaban subgroup odds ratio increased from 0.83 [0.20, 3.50] (p = 0.80) to 1.08 [0.62, 1.88] (p = 0.80). Addition of AMPLIFY which lacked any fatal bleeding events increased the odds ratio minimally to 0.97 [0.71,1.33] (p = 0.85).

**Fig 5:**
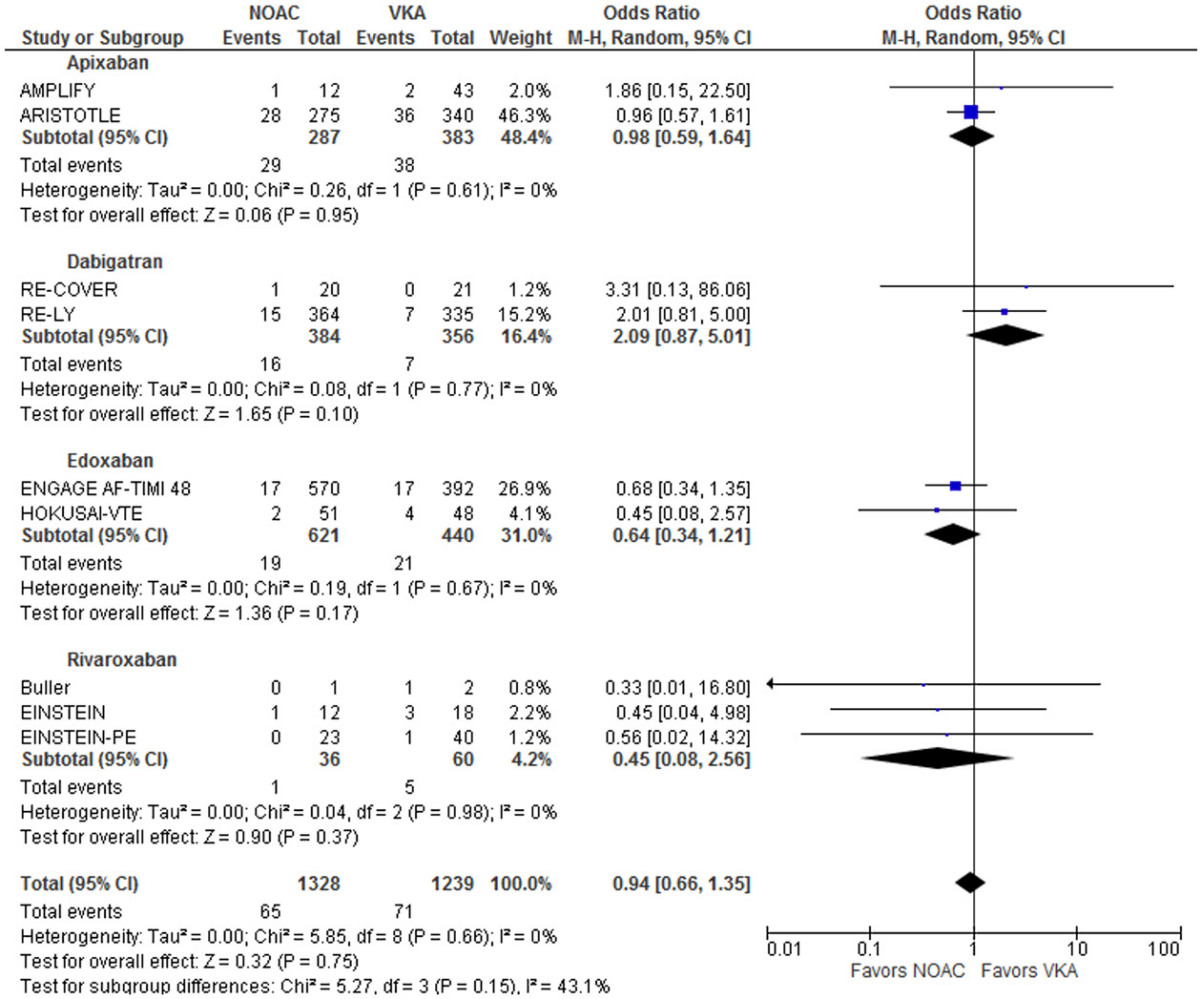
Relative odds of fatal bleeding given patient with major extracranial bleeding event. Odds ratios and 95% confidence intervals are given for the relative odds that major extracranial bleeding events will lead to fatal bleeding events.

Nine studies had data available on the risk of death in extracranial bleeding. In total, 2567 patients experienced at least one extracranial bleed. 136 bleeds resulted in death. The total odds ratio for fatal bleeding given an extracranial bleed occurred was 0.94 [0.66, 1.35] (p = 0.75) (Fig 5). This result was not statistically significant. The effect sizes were homogeneous across all the included studies with an I^2^ value of 0%. Homogeneity was retained when comparing individual medication classes. Testing for subclass heterogeneity resulted in an I^2^ value of 43.1% (P = 0.15).

Sensitivity analysis was performed for extracranial bleeding episodes. Adjustment for total major bleeding events in nine studies had no effect on the total odds ratio which remained at 0.94 [0.66, 1.34] (p = 0.73) (S7 Fig). Addition of pooled results from five studies with extracranial bleeds in only one trial arm minimally reduced the odds ratio to 0.93 [0.65, 1.32] (p = 0.68). Adjustment for multiple treatment arms in ENGAGE AF-TIMI 48 minimally increased the odds ratio to 0.95 [0.67, 1.36] (p = 0.79). Adjustment for zero cells in four studies increased the odds ratio minimally to 0.96 [0.68, 1.35] (p = 0.79).

## Discussion

### Summary of Main Results

This meta-analysis provides a summary ratio for the conditional odds of fatal bleeding in patients with major bleeds provoked by NOAC compared to VKA use. Data was synthesized from all studies done to date of NOACs compared to VKAs for thromboembolism prophylaxis in atrial fibrillation and acute venous thromboembolism.

The frequency of major bleeding with NOAC and VKA agents has been detailed elsewhere [36, 37]. The focus of this analysis was to explore the outcomes of major bleeds once they occurred. Taken as a class, the NOACs showed reduced odds of death following major bleeds. However, in the subpopulations of intracranial and extracranial bleeds, there was no statistically significant difference detected in odds of death between bleeds occurring on NOACs versus VKAs. This comparison of a specific site of bleeding suggests that there is no evidence for an intrinsic difference in the risk of death from major bleeds but that the composition of bleeding sites in NOAC and VKA associated bleeding is different. Indeed, intracranial bleeding occurs twice as frequently with warfarin as with the NOAC agents [37]. In addition, intracranial bleeds are fatal far more often than extracranial bleeds. In our study, despite intracranial bleeding accounting for only 18% of major bleeds, 78% of all fatal bleeds were intracranial. Rather than detect any underlying difference in severity of bleeding associated with NOAC versus VKA use, our study reemphasizes the increased tendency of VKAs to cause bleeding in sites with higher mortality risk that has been repeatedly shown in phase III NOAC trials. One potential mechanism for increased risk of intracerebral hemorrhage with VKAs has been proposed. Tissue factor and factor VIIa complexes play a major role in hemostasis during intracranial bleeds. Warfarin inhibits factor VIIa formation while the direct thrombin and factor Xa inhibitors do not. [38]

The results of this study were robust. Adjustment for the indication for anticoagulation, atrial fibrillation or venous thromboembolism, did not reveal any heterogeneity in the results from these two subgroups. Despite the significant demographic differences between these two subpopulations, the reduced risk of death in major bleeding on NOACs occurred to a similar degree in both groups. Similar analysis with blinded and unblinded studies also showed no heterogeneity. There were several approximation methods used in this study to calculate the results and were detailed above. In the sensitivity analysis, results calculated from different methods of approximation were compared and there was no clinically significant variation in the odds ratios.

An advantage to this study was the use of randomized cohorts. The results are less likely influenced by confounding demographic differences between populations assigned to NOAC or VKA treatment. Limited data has been published on the actual baseline characteristics of the subset of patients with bleeds in the included trials. In the ARISTOTLE trial [19], patients with major bleeds on NOACs and VKAs were well matched with regard to all baseline characteristics examined, including age, gender, CHADS2 score, HASBLED score and dual antiplatelet use. Baseline characteristics were also well matched in the ROCKET-AF trial (S2 Table). However, different baseline characteristics may predispose patients to bleeding on NOACs versus bleeding on VKAs and create heterogeneity between the populations compared in this study. This occurred in the trials of dabigatran [39]. While there were no clinically significant differences between the groups in regards to gender, weight, aspirin or NSAID use, mean age was 75.1 versus 71.8 years (p<0.0001) and creatinine clearance was 55 versus 62 mL/min (p<0.0001) for the dabigatran and warfarin groups, respectively. Both age and renal impairment are associated with worse outcomes so adjustment for these imbalances would likely create a shift toward better outcomes with dabigatran relative to VKA associated bleeds. Such analysis should be explored in future studies but given the data currently available, the homogeneity between study groups created by randomization appears to be preserved. This is a major strength of this study. Future studies that prospectively evaluate the protocolized management of NOAC and VKA associated bleeding can better compare bleeding outcomes under optimal treatment conditions. However, these studies will not be randomized and will suffer from confounding variables that can only be adjusted for statistically.

A significant confounding factor in the data analyzed in this study is the variable approaches to managing bleeding events. Because there is no high level of evidence to direct the management of NOAC associated bleeding, there was no specific protocol for anticoagulation reversal devised in any of the included studies. All studies had 24-hour telephone hotlines to contact for suggestions on management of bleeds. The treatment code was not routinely broken. Protocols generally recommended three steps in bleeding management. First, discontinue or delay the next dose of the study drug. Second, use supportive measures such as intravenous fluid and blood product replacement if indicated to maintain perfusion. Third, administer vitamin k and fresh frozen plasma as the patient may be assigned to a VKA followed by procoagulants such as rFVIIa or three or four factor complex concentrates if bleeding remains uncontrolled. There were no specific thresholds indicated in the protocols for when to administer the treatments, instead clinical judgement was encouraged. Of the included studies, only the phase III dabigatran trials [39] have examined in detail the management of bleeding events. Major bleeds were primarily treated with supportive measures. Blood product replacement was administered in 61.4% and 49.9% (p<0.001), vitamin K in 10.3% and 27.3% (p<0.001), fresh frozen plasma in 21.6% and 30.2% (p = 0.005), hospitalization in 61.8% and 56.5% (p = 0.01) and surgery in 14.0% and 15.0% (p = 0.76) of dabigatran and VKA associated bleeds, respectively. Procoagulants were seldom used. rFVIIa was used in only 1.8% and 0.7% of dabigatran and VKA associated bleeds, respectively. Similar numbers for PCC use were 0.5% and 1.2%. There were significant differences in the approach to managing bleeds on dabigatran versus VKAs. This may relate to the open label design of the RE-LY trial. The double-blinded ARISTOTLE study has also analyzed transfusion use in major bleeds and the rate was similar at 37.8% and 37.0% in the apixaban and VKA arms, respectively. [40]

As the optimal management strategy for NOAC associated bleeding becomes better defined, data from these trials should be reexamined to determine the degree to which variable use of treatments for bleeds on NOACs versus VKAs influenced the relative fatal outcomes. Clinicians should interpret the results of this study in the appropriate context. The findings best apply to clinical settings similar to those of the included studies in which management of bleeding was based largely on the providers’ best clinical judgement and no specific threshold was used to trigger certain treatments. Supportive measures such as blood transfusion and hospitalization were commonly used, fresh frozen plasma and surgery were less frequently used and procoagulant factors were seldom used. The studies generally excluded patients with relative contraindications to anticoagulation which would resultin higher than average baseline bleeding risks. In the appropriate clinical setting, these findings suggest that bleeding outcomes at a given anatomical site are likely to be similar whether the patient was treated with a NOAC agent or VKA.

### Quality of the Evidence

Overall the results of the included studies were at low risk of bias. Sequence generation, allocation, blinding and completeness of outcome were adequate in all but two trials included in the primary analysis. In the J-ROCKET AF trial, methodology for sequence generation and allocation was not reported. In the RE-LY trial, early withdrawals were disproportionately high in the dabigatran arm at 21% versus 16.6% of patients withdrawing early in the control arm. Blinding was judged adequate in all studies. Several studies were open label for the participants and care providers. This was felt to be a low risk for bias as major bleeding events is an objective outcome and is unlikely to be influenced by the clinician’s or patient’s knowledge of treatment assignment. There was no difference in treatment effect size for double-blinded compared to open label studies.

In all studies, the outcomes were assessed by a blinded third party. This blinding strengthened the studies as determination of fatal bleeding is subjective. Unlike major bleeding which is determined by meeting one of three objective criteria (fall of hemoglobin more than 2 g/dL, transfusion of more than 2 units, location in a critical organ), there were no prespecified objective criteria for determining whether a bleed was fatal. While death is clearly objective, determining whether the death was specifically caused by the underlying bleed is not. ENGAGE-AF TIMI 48 eliminated some subjectivity by limiting fatal bleeding events to deaths that occurred within 7 days of the bleed. Given this subjective limitation, the blinding of the events committees in all included studies was a major strength.

### Study Limitations

It was not possible to calculate the odds ratio for death in extracranial bleeding for all studies included in this review. ENGAGE-AF TIMI 48, HOKUSAI-VTE and RE-COVER did not analyze extracranial bleeding deaths. Extracranial bleeding was estimated for these studies by determining the number of patients with a major bleed and no intracranial bleed. This estimate is precise when there are no patients with both extracranial and intracranial bleeding. Even when this assumption is false, the number of patients with both intracranial and extracranial bleeding is relatively small and the error introduced by this assumption is minimal. J-ROCKET AF, RE-COVER II AND RE-MEDY had no fatal extracranial bleeds and were excluded from the primary extracranial bleeding analysis. Inclusion of these studies did not lead to a clinically significant change in the results. ROCKET AF was excluded from this analysis as well because data on major and intracranial bleeding were collected from separate published reports. From these reports, intracranial bleeding data included all bleeds during follow-up while the major bleeding data included only bleeding events occurring while on treatment.

The literature search for this systematic review was undertaken by a single reviewer. This approach was chosen as multiple other systematic reviews had already been published that examine different clinical questions with this same group of clinical trials [37, 41–44]. The studies included in these reviews matched well with the list of included studies from this review. This external validity was judged sufficient to proceed without a second reviewer repeating the literature search.

## Authors' Conclusions

Major bleeds occurring on NOAC therapy are statistically significantly less likely to lead to death than bleeds occurring with VKAs. This reduction in fatal bleeding is due to the decreased incidence of intracranial bleeding with NOAC agents compared to VKAs. Odds of fatal bleeding given occurrence of a major bleed at any given anatomic site showed no detectable difference between NOAC and VKA groups. Despite poor understanding of anticoagulation reversal with the NOAC agents, there is no additional mortality risk detected during bleeding events compared to VKAs.

## Supporting Information

S1 Doc PROSPERO protocol. Protocol registered with PROSPERO detailing the methods used for the systematic review and meta-analysis.
(PDF)

S2 Doc Meta-analysis data set. Source data for all analyses performed within the study. Individual studies listed in rows. Additional rows contain data for alternative methods for calculating the number of bleeds. Individual outcomes listed in columns.
(XLSX)

S1 Fig Characteristics of studies and assessment of risk of bias. (PDF)

S2 Fig Summary of risk of bias in individual studies. Color code green = low risk for bias, yellow = unclear risk and red = high risk.
(TIF)

S3 Fig Risk of bias graph. Percentages represent the proportion of studies with each level of risk for bias in each of the domains.
(TIF)

S4 Fig Relative odds of fatal bleed in double-blinded and open label subgroups. Odds ratios and 95% confidence intervals are given for the relative odds that major bleeding events will lead to fatal bleeding events. Subgroups are determined by the methods for blinding used in the included studies and are divided into double-blind and open label studies.
(TIF)

S5 Fig Relative odds of fatal bleed given occurrence of major bleed-adjustment for total bleeds. Odds ratios and 95% confidence intervals are given for the relative odds that major bleeding events will lead to fatal bleeding events. Calculations were made using the number of total bleeding events for the following studies: EINSTEIN, EINSTEIN-PE, ENGAGE AF-TIMI 48, HOKUSAI-VTE, J-ROCKET, RE-COVER, RE-COVER II, RE-LY AND RE-MEDY.
(TIF)

S6 Fig Relative odds of fatal bleed given occurrence of major bleed-adjustment for multiple treatment arms. Odds ratios and 95% confidence intervals are given for the relative odds that major bleeding events will lead to fatal bleeding events. Calculations were made using the events from the multiple treatment arms of ENGAGE AF-TIMI 48 split among two trials and the events of the VKA control split in proportion to the size of the treatment groups.
(TIF)

S7 Fig Relative odds of fatal bleed given occurrence of major extracranial bleed-adjustment for total bleeding events. Odds ratios and 95% confidence intervals are given for the relative odds that major extracranial bleeding events will lead to fatal bleeding events. Calculations were made using the number of total bleeding events for the following studies: EINSTEIN, EINSTEIN-PE, ENGAGE AF-TIMI 48, HOKUSAI-VTE, J-ROCKET, RE-COVER, RE-COVER II, RE-LY AND RE-MEDY.
(TIF)

S8 Fig Cochrane search strategy. Example of the search strategy used to systematically review the studies indexed in the Cochrane database.
(PDF)

S9 Fig PRISMA checklist. Checklist of PRISMA criteria and location within article.
(PDF)

S1 Table Data extraction sheet. Tool used to extract data from included studies.
(PDF)

S2 Table Baseline characteristics of patients with bleed in ROCKET AF. Values are percentages of patients in each treatment arm with the individual characteristics at baseline. Fisher’s exact test was used to calculate p-values.
(PDF)
