# Supplementary material for: Risk of Fatal Bleeding in Episodes of Major Bleeding with New Oral Anticoagulants and Vitamin K Antagonists: A Systematic Review and Meta-Analysis
Source: PLoS One. 2015 Sep 18;10(9):e0137444. doi: 10.1371/journal.pone.0137444 (PMC4575170; doi:10.1371/journal.pone.0137444)
Supplement: S1 Doc — Protocol registered with PROSPERO detailing the methods used for the systematic review and meta-analysis. (PDF) [file pone.0137444.s001.pdf]

Risk of bleeding-related fatalities with reversible and non-reversible oral anticoagulants: a systematic review and meta-analysis

Joel Skaistis

Citation

Joel Skaistis. Risk of bleeding-related fatalities with reversible and non-reversible oral anticoagulants: a systematic review and meta-analysis. PROSPERO 2014:CRD42014013294 Available from
http://www.crd.york.ac.uk/PROSPERO_REBRANDING/display_record.asp?ID=CRD42014013294

Review question(s)

Is there a difference in the conditional probability of death given a major bleed has occurred for patients treated with non-reversible oral anticoagulants versus reversible oral anticoagulants?

Is there a difference in the conditional probability of death given a major gastrointestinal bleed has occurred for patients treated with non-reversible oral anticoagulants versus reversible oral anticoagulants?

Is there a difference in the conditional probability of death given a major intracranial bleed has occurred for patients treated with non-reversible oral anticoagulants versus reversible oral anticoagulants?

Searches

The following databases will be searched: PubMed, Cochrane Central Register of Controlled Trials (CENTRAL), Ovidweb, Clinicaltrial.gov register, ISRCTN register, World Health Organization ICTRP register. These databases will be searched with the following MeSH terms, keywords and limits:

Population: pulmonary embolism, atrial fibrillation, stroke, venous thromboembolism, embolism, embolism and thrombosis

Intervention: dabigatran, edoxaban, rivaroxaban, apixaban

Study design: Randomized control trial

Types of study to be included

Inclusions: randomized controlled trials that evaluated major bleeding as a primary or secondary outcome.

Exclusions: pharmacokinetic studies, studies without major bleeding events

Condition or domain being studied

Diseases for which anticoagulants are prescribed will be studied. This will include specifically, pulmonary embolism, atrial fibrillation, stroke, venous thromboembolism, embolism, embolism and thrombosis

Intervention.

Participants/ population

Inclusions: Patients treated with oral anticoagulation for thromboembolic treatment or prophylaxis and were enrolled in a trial comparing vitamin K antagonist therapy to non-vitamin k antagonist therapy anticoagulation; age > 18; experienced major bleeds while on anticoagulation.

Exclusions: none

Intervention(s), exposure(s)

Inclusions: non-reversible oral anticoagulant limited to dabigatran 150 mg twice daily, rivaroxaban 20 mg daily,

apixaban 2.5 mg twice daily or 5 mg twice daily, edoxaban 30 mg daily or 60 mg daily.

Exclusions: Drug dosages other than those listed above were excluded as they are not used in clinical practice.

Comparator(s)/ control
Comparators will be limited to vitamin K antagonists or vitamin k antagonists bridged with heparin.

Outcome(s)
Primary outcomes
Major bleed events, fatal bleed events

Secondary outcomes
Major gastrointestinal bleed events, fatal gastrointestinal bleed events, major intracranial bleed events, fatal intracranial bleed events

Data extraction, (selection and coding)
A single reviewer will extract data according to a data extraction sheet. Extraction will be performed twice to facilitate accuracy. Authors of studies will be contacted to provide missing data. The following data will be extracted: Trial; Indication (VTE, AFIB, etc.); Exclusions; Study type; Sequence generation (1=low risk, 0=unclear, -1=high risk); Allocation concealment (1,0,-1); Blinding (1,0,-1); Incomplete outcome data (1,0,-1); Intervention (I); Control (C); PMID/NCT; primary data=1; duplicate data=0; Bleed definition (ISTH v. other); I withdrawal %; C withdrawal %; I study size; C study size; I age; C age; I % age > 75; C % age > 75; I % GFR<50; C % GFR<50; I % previous CVA/TIA; C % previous CVA/TIA; I % antiplatelet use; C % antiplatelet use; I major bleeds #; C major bleeds #; I fatal bleeds #; C fatal bleeds #; I non-fatal major bleeds #; C non-fatal major bleeds #; I major gastrointestinal bleeds #; C major gastrointestinal bleeds #; I fatal GI bleed #; C fatal GI bleed #; I major intracranial bleeds #; C major intracranial bleeds #; I fatal intracranial bleeds #; C intracranial bleeds #; I non-CNS major bleed #; C non-CNS major bleed #

Risk of bias (quality) assessment
Study validity will be assessed with the Cochrane Collaboration's risk of bias assessment tool. The following domains will be evaluated: sequence generation, allocation concealment, blinding, incomplete outcome data. Study quality assessments will be used for sensitivity analysis.

Strategy for data synthesis
Aggregate data extracted from trials will be combined by a random effects model with effect sizes given by odds ratios calculated by the Mantel-Haenszel method. Heterogeneity will be evaluated with I-squared calculations. Statistical analysis will be performed with RevMan 5.3 with two tailed p-values <0.05 considered significant.

Analysis of subgroups or subsets
Drug type will be used in subgroup analysis.

Contact details for further information
Joel Skaistis

19491 Eddington Pl

Northville, MI

48167

joeleth@gmail.com

Organisational affiliation of the review
William Beaumont Hospital

Review team

Dr Joel Skaistis,

Anticipated or actual start date
18 August 2014

Anticipated completion date
30 September 2014

Funding sources/sponsors
No external sources were used to finance this review. Joel Skaistis has no conflicts of interests to declare.

Conflicts of interest
None known

Language
English

Country
United States of America

Subject index terms status
Subject indexing assigned by CRD

Subject index terms
Anticoagulants; Hemorrhage; Humans

Stage of review
Ongoing

Date of registration in PROSPERO
18 August 2014

Date of publication of this revision
18 August 2014

**Stage of review at time of this submission**	**Started**	**Completed**
Preliminary searches	Yes	No
Piloting of the study selection process	Yes	No
Formal screening of search results against eligibility criteria	Yes	No
Data extraction	No	No
Risk of bias (quality) assessment	Yes	No
Data analysis	No	No

PROSPERO
International prospective register of systematic reviews
The information in this record has been provided by the named contact for this review. CRD has accepted this information in good faith and registered the review in PROSPERO. CRD bears no responsibility or liability for the content of this registration record, any associated files or external websites.
