# Supplementary material for: Risk of Fatal Bleeding in Episodes of Major Bleeding with New Oral Anticoagulants and Vitamin K Antagonists: A Systematic Review and Meta-Analysis
Source: PLoS One. 2015 Sep 18;10(9):e0137444. doi: 10.1371/journal.pone.0137444 (PMC4575170; doi:10.1371/journal.pone.0137444)
Supplement: S1 Fig — (PDF) [file pone.0137444.s003.pdf]

	Methods	Participants	Interventions	Outcomes	Random sequence generation (selection bias)	Allocation concealment (selection bias)	Blinding of participants and personnel (performance bias)	Blinding of outcome assessment (detection bias)	Incomplete outcome data (attrition bias)	Selective reporting (reporting bias)
**Agnelli**	RCT double-blind treatment arm, open label control	Patients with DVT. Excluded CVA, intracranial or gastrointestinal bleed in < 6 months; active gastric ulcer; CrCl<30; LFTs > 2x ULN	Rivaroxaban 10 mg BID versus AD VKA	ISTH major bleeds, fatal bleeds	Low risk	Low risk	Low risk	Low risk	Low risk	Low risk
					Randomization by centralized computer.	Randomization by centralized computer.	Patients and providers unblinded to treatment but outcome assessors were blinded and given objective nature of outcomes (major bleed, death), unlikely lack of blinding would introduce bias.	Blinded central adjudication committee assessed outcomes.	Comparable early withdrawals across arms.	Not applicable given specific focus on bleeding outcomes in this meta-analysis.
**AMPLIFY**	RCT, double-blind, triple-dummy	Patients with acute VTE, excluding those with: high risk of bleeding; absence of risk factors for recurrent VTE; dual-antiplatelet therapy; aspririn use > 165 mg; Hemoglobin<9 mg/dL; platelet < 100,000/cubic cm; CrCl<25 ml/min.	Apixaban 5 mg BID; 2.5 mg BID if age>80, weight<60 kg, Cr>1.5 mg/dL versus AD VKA (adjusted dose vitamin-K antagonist)	ISTH major bleeds, fatal bleeds, extracranial bleeds	Low risk	Low risk	Low risk	Low risk	Low risk	Low risk
					Randomization by centralized computer.	Randomization by centralized computer.	Patients and providers blinded. Sham INR measurements used.	Blinded central adjudication committee assessed outcomes.	Comparable early withdrawals across arms. Treatment: 13.3% Placebo: 14.4%.	Not applicable given specific focus on bleeding outcomes in this meta-analysis.
**ARISTOTLE**	RCT, double-blind, double-dummy	Patients with AFIB, excluding those with: CVA within previous 7 days; aspirin > 165 mg/day; dual-antiplatelet therapy; CcCl<25 mL/min	Apixaban 5 mg BID; 2.5 mg BID if age>80, weight<60 kg, Cr>1.5 mg/dL versus AD VKA	ISTH major bleeds, fatal bleeds, intracranial bleeds, extracranial bleeds,	Low risk	Low risk	Low risk	Low risk	Low risk	Low risk
					Randomization by centralized computer.	Randomization by centralized computer.	Patients, providers and outcome assessors all binded. Sham INR measurements used.	Blinded central adjudication committee assessed outcomes.	Comparable early withdrawals across arms. Treatment: 21.7% Control: 23.7%.	Not applicable given specific focus on bleeding outcomes in this meta-analysis.
**ARISTOTLE-J**	RCT, double blind treatment arm, open label control	Patients with AFIB. Excluded recent CVA/TIA; ASA>100; recent major surgery; ulcer; AST/ALT>2 ULN; CrCL<25	Apixaban 5 mg BID versus AD VKA	ISTH major bleeds, fatal bleeds	Unclear risk	Unclear risk	Low risk	Low risk	Unclear risk	Unclear risk
					Not reported.	Not reported.	Patients and providers unblinded to treatment but outcome assessors were blinded and given objective nature of outcomes (major bleed, death), unlikely lack of blinding would introduce bias.	Blinded central adjudication committee assessed outcomes.	Not reported.	Not applicable given specific focus on bleeding outcomes in this meta-analysis.
**BOTICELLI**	RCT, single-arm double blind	Patients with DVT. Excluded liver dysfunction, "high risk for bleeding" BP>200/100, ASA>165	Apixaban 5 mg BID versus AD VKA	ISTH major bleeds, fatal bleeds	Low risk	Low risk	Low risk	Low risk	Low risk	Low risk
					Randomization by centralized computer.	Randomization by centralized computer.	Patients and providers unblinded to treatment but outcome assessors were blinded and given objective nature of outcomes (major bleed, death), unlikely lack of blinding would introduce bias.	Blinded central adjudication committee assessed outcomes.	Comparable early withdrawals across arms.	Not applicable given specific focus on bleeding outcomes in this meta-analysis.
**Buller**	RCT, double-blind treatment arm, open label control	Patients with acute DVT, excluding those with: high risk of bleeding; BP>200/110 mmHg; CrCl<30 m:/min; ALT<2x ULN;	Rivaroxaban 15 mg BID x 3 weeks then 20 mg qday versus AD VKA	ISTH major bleeds, fatal bleeds	Low risk	Low risk	Low risk	Low risk	Low risk	Low risk
					Randomization by centralized computer.	Randomization by centralized computer.	Patients and providers unblinded to treatment but outcome assessors were blinded and given objective nature of outcomes (major bleed, death), unlikely lack of blinding would introduce bias.	Blinded central adjudication committee assessed outcomes.	Comparable early withdrawals across arms.	Not applicable given specific focus on bleeding outcomes in this meta-analysis.

	Methods	Participants	Interventions	Outcomes	Random sequence generation (selection bias)	Allocation concealment (selection bias)	Blinding of participants and personnel (performance bias)	Blinding of outcome assessment (detection bias)	Incomplete outcome data (attrition bias)	Selective reporting (reporting bias)
**Chung**	RCT, double-blind treatment arm, open label warfarin	Patients with AFIB. Excluded past history of major bleeding; uncontrolled hypertension; uncontrolled diabetes; hemorrhagic disorder; significant thrombocytopenia; LFTs >2 ULN; Cr>1.5	Edoxaban 60 mg qday, edoxaban 30 mg qday versus AD VKA		Low risk	Low risk	Low risk	Low risk	Low risk	Low risk
					Randomization by centralized computer.	Randomization by centralized computer.	Patients and providers unblinded to treatment but outcome assessors were blinded and given objective nature of outcomes (major bleed, death), unlikely lack of blinding would introduce bias.	Blinded central adjudication committee assessed outcomes.	Comparable early withdrawals across arms.	Not applicable given specific focus on bleeding outcomes in this meta-analysis.
**EINSTEIN**	RCT, open label	Patients with acute DVT, excluding those with:CrCl<30 mL/min; clinically significant liver disease; LFTs>3x ULN; high risk of bleeding; BP>180/110 mmHg;	Rivaroxaban 15 mg BID x 3 weeks then 20 mg qday versus AD VKA	ISTH major bleeds, fatal bleeds, intracranial bleeds, extracranial bleeds	Low risk	Low risk	Low risk	Low risk	Low risk	Low risk
					Randomization by centralized computer.	Randomization by centralized computer.	Patients and providers unblinded to treatment but outcome assessors were blinded and given objective nature of outcomes (major bleed, death), unlikely lack of blinding would introduce bias.	Blinded central adjudication committee assessed outcomes.	Comparable early withdrawals across arms. Treatment: 11.3% Control: 14.2%.	Not applicable given specific focus on bleeding outcomes in this meta-analysis.
**EINSTEIN-PE**	RCT, open label	Patients with acute PE, excluding those with: CrCl<30 mL/min; significant liver disease; LFTs>3x ULN; high bleeding risk; BP>180/110 mmHg	Rivaroxaban 15 mg BID x 3 weeks then 20 mg qday versus AD VKA	ISTH major bleeds, fatal bleeds, intracranial bleeds, extracranial bleeds	Low risk	Low risk	Low risk	Low risk	Low risk	Low risk
					Randomization by centralized computer.	Randomization by centralized computer.	Patients and providers unblinded to treatment but outcome assessors were blinded and given objective nature of outcomes (major bleed, death), unlikely lack of blinding would introduce bias.	Blinded central adjudication committee assessed outcomes.	Comparable early withdrawals across arms. Treatment: 10.7% Control: 12.3%.	Not applicable given specific focus on bleeding outcomes in this meta-analysis.
**ENGAGE AF-TIMI 48**	RCT, double-blind, double-dummy	Patients with AFIB, excluding those with: dual antiplatelet therapy; high risk of bleeding; CrCl<30 mL/min; CVA < 30 days prior	Edoxaban 60 mg qday versus AD VKA	ISTH major bleeds, fatal bleeds, intracranial bleeds, extracranial bleeds	Low risk	Low risk	Low risk	Low risk	Low risk	Low risk
					Randomization by centralized computer.	Randomization by centralized computer.		Blinded central adjudication committee assessed outcomes.	Comparable early withdrawals across arms. Treatment: 25.1% Control: 24.5%.	Not applicable given specific focus on bleeding outcomes in this meta-analysis.
**HOKUSAI-VTE**	RCT, double-blind, double-dummy	Patients with acute VTE, excluding those with: ASA>100 mg/day; CrCl < 30 mL/min	Open label low molecular weight heparin followed by Edoxaban 60 mg qday or 30 mg qday for CrCl<50 mL/min or weight<60 kg versus AD VKA	ISTH major bleeds, fatal bleeds, intracranial bleeds, extracranial bleeds	Low risk	Low risk	Low risk	Low risk	Low risk	Low risk
					Randomization by centralized computer.	Randomization by centralized computer.	Patients, providers and outcome assessors all binded. Sham INR measurements used.	Blinded central adjudication committee assessed outcomes.	Comparable early withdrawals across arms. Treatment: 1.8% Control: 1.7%.	Not applicable given specific focus on bleeding outcomes in this meta-analysis.
**NCT01136408**	RCT, double open label	Patients with AFIB. Excluded CVA <30 days; major non-GI bleeding in < 6 months; uncontrolled hypertension	Dabigatran 150 mg BID versus AD VKA	ISTH major bleeds, fatal bleeds	Unclear risk	Unclear risk	Low risk	Low risk	High risk	Low risk

	Methods	Participants	Interventions	Outcomes	Random sequence generation (selection bias)	Allocation concealment (selection bias)	Blinding of participants and personnel (performance bias)	Blinding of outcome assessment (detection bias)	Incomplete outcome data (attrition bias)	Selective reporting (reporting bias)
					Not stated.	Not stated.	Patients and providers unblinded to treatment but outcome assessors were blinded and given objective nature of outcomes (major bleed, death), unlikely lack of blinding would introduce bias.	Blinded central adjudication committee assessed outcomes.	Not balanced between arms. 15.5% in dabigatran arm, 8.0% in VKA control.	Not applicable given specific focus on bleeding outcomes in this meta-analysis.
**J-ROCKET AF**	RCT double-blind, double-dummy	Patients with AFIB, excluding those with: major surgery or trauma < 30 days prior; major gastrointestinal bleed in < 6 months; history of intracranial, intraocular, spinal or atraumatic intra-articular bleeding; platelet < 90,000/uL; BP>180/100 mmHg; CVA < 3 months; Aspirin > 100 mg/day; CrCL< 30; LFTs>3x ULN	Rivaroxaban 15 mg qday versus AD VKA	ISTH major bleeds, fatal bleeds, intracranial bleeds	Unclear risk	Unclear risk	Low risk	Low risk	Low risk	Low risk
					Not stated.	Not stated.	Patients, providers and outcome assessors all binded. Sham INR measurements used.	Blinded central adjudication committee assessed outcomes.	Comparable early withdrawals across arms. Treatment: 13.1% Control: 15%.	Not applicable given specific focus on bleeding outcomes in this meta-analysis.
**RE-COVER**	RCT, double blind, double dummy	Patients with acute VTE, excluding those with: high-risk of bleeding; AST/ALT>2x ULN; Cr Cl<30 mL/min; Aspirin>100 mg/day	Dabigatran 150 mg BID versus AD VKA	ISTH major bleeds, fatal bleeds, extracranial bleeds	Low risk	Low risk	Low risk	Low risk	Low risk	Low risk
					Randomization by centralized computer.	Randomization by centralized computer.	Patients, providers and outcome assessors all binded. Sham INR measurements used.	Blinded central adjudication committee assessed outcomes.	Comparable early withdrawals across arms. Treatment: 16% Control: 14.5%.	Not applicable given specific focus on bleeding outcomes in this meta-analysis.
**RE-COVER II**	RCT, double blind, double dummy	Patients with acute VTE, excluding those with: high-risk of bleeding; AST/ALT>2x ULN; Cr Cl<30 mL/min; Aspirin>100 mg/day	Dabigatran 150 mg BID versus AD VKA	ISTH major bleeds, fatal bleeds, intracranial bleeds	Low risk	Low risk	Low risk	Low risk	Low risk	Low risk
					Randomization by centralized computer.	Randomization by centralized computer.	Patients, providers and outcome assessors all binded. Sham INR measurements used.	Blinded central adjudication committee assessed outcomes.	Comparable early withdrawals across arms. Treatment: 14.7% Control: 14.1%.	Not applicable given specific focus on bleeding outcomes in this meta-analysis.
**RE-LY**	RCT, double blind treatment arm, open label control	Patients with AFIB, excluding those with surgery < 1 month prior; any critical organ bleeding; gastrointestinal hemorrhage in < 1 year; gastroduodenal ulcer in < 30 days; BP>180/100 mmHg; CrCL<30 mL/min; AST/ALT>2x ULN	Dabigatran 150 mg BID versus AD VKA	ISTH major bleeds, fatal bleeds, intracranial bleeds, extracranial bleeds	Low risk	Low risk	Low risk	Low risk	High risk	Low risk
					Randomization by centralized computer.	Randomization by centralized computer.	Patients and providers unblinded to treatment but outcome assessors were blinded and given objective nature of outcomes (major bleed, death), unlikely lack of blinding would introduce bias.	Blinded central adjudication committee assessed outcomes.	Significant higher early withdrawals in treatment arm. Treatment: 21.2%. Control: 16.6%. p<0.0001	Not applicable given specific focus on bleeding outcomes in this meta-analysis.
**RE-MEDY**	RCT, double blind, double dummy	Patients with VTE previously for 3-12 months duration, excluding those with: active VTE; trauma/surgery in past month; history of critical site bleeding; Gastrointestinal bleed < 3 months prior; Hgb<10 g/dL; CrCL<30 mL/min; AST/ALT>2x ULN	Dabigatran 150 mg BID versus AD VKA	ISTH major bleeds, fatal bleeds, intracranial bleeds	Low risk	Low risk	Low risk	Low risk	Low risk	Low risk
					Randomization by centralized computer.	Randomization by centralized computer.	Patients, providers and outcome assessors all binded. Sham INR measurements used.	Blinded central adjudication committee assessed outcomes.	Comparable early withdrawals across arms. Treatment: 19.3% Control: 19.7%.	Not applicable given specific focus on bleeding outcomes in this meta-analysis.

	Methods	Participants	Interventions	Outcomes	Random sequence generation (selection bias)	Allocation concealment (selection bias)	Blinding of participants and personnel (performance bias)	Blinding of outcome assessment (detection bias)	Incomplete outcome data (attrition bias)	Selective reporting (reporting bias)
**ROCKET AF**	RCT, double-blind, double-dummy	Patients with AFIB, excluding those with: major bleed < 30 days prior; hemodynamically significant gastrointestinal bleed < 6 months prior; history of critical organ bleeding; platelet< 90,000/uL; BP>180/100; severe CVA < 3 months prior; Aspirin>100 mg/day; dual-antiplatelet therapy; Hemoglobin<10 g/dL; CrCL<30 mL/min; LFTs>3x ULN	Rivaroxaban 20 mg qday versus AD VKA	ISTH major bleeds, fatal bleeds, intracranial bleeds	Low risk	Low risk	Low risk	Low risk	Low risk	Low risk
					Randomization by centralized computer.	Randomization by centralized computer.	Patients, providers and outcome assessors all binded. Sham INR measurements used.	Blinded central adjudication committee assessed outcomes.	Comparable early withdrawals across arms. Treatment: 18.2% Control: 16.9%.	Not applicable given specific focus on bleeding outcomes in this meta-analysis.
**Weitz**	RCT, double-blind, double-dummy treatment arm, open label warfarin	Patients with AFIB, excluded recent major bleeding; uncontrolled hypertension; hemoglobin<10; platelet<100,000/uL; LFTs > 1.5x ULN; Bilirubin>ULN; CrCl<30;	Edoxaban 60 mg qday, edoxaban 30 mg qday versus AD VKA	ISTH major bleeds, fatal bleeds	Low risk	Low risk	Low risk	Low risk	Unclear risk	Low risk
					Randomization by centralized computer.	Randomization by centralized computer.	Patients and providers unblinded to treatment but outcome assessors were blinded and given objective nature of outcomes (major bleed, death), unlikely lack of blinding would introduce bias.	Blinded central adjudication committee assessed outcomes.	Not reported	Not applicable given specific focus on bleeding outcomes in this meta-analysis.
**Yamashita**	RCT, double-blind treatment arm, open label warfarin	Patients with AFIB, excluded history of critical organ bleeding; gastrointestinal bleeding in < 1 year; hemoglobin < 10 g/dl; Platelet<100/uL; CVA < 30 days; concurrent anticoagulant treatment	edoxaban 60 qd, edoxaban 30 qd versus AD VKA	ISTH major bleeds, fatal bleeds	Low risk	Unclear risk	Low risk	Low risk	Low risk	Low risk
					Randomization by centralized computer.	Not reported.	Patients and providers unblinded to treatment but outcome assessors were blinded and given objective nature of outcomes (major bleed, death), unlikely lack of blinding would introduce bias.	Blinded central adjudication committee assessed outcomes.	Comparable early withdrawals across arms.	Not applicable given specific focus on bleeding outcomes in this meta-analysis.
