# Supplementary figures and images for: Risk of Fatal Bleeding in Episodes of Major Bleeding with New Oral Anticoagulants and Vitamin K Antagonists: A Systematic Review and Meta-Analysis
Source: PLoS One. 2015 Sep 18;10(9):e0137444. doi: 10.1371/journal.pone.0137444 (PMC4575170; doi:10.1371/journal.pone.0137444)

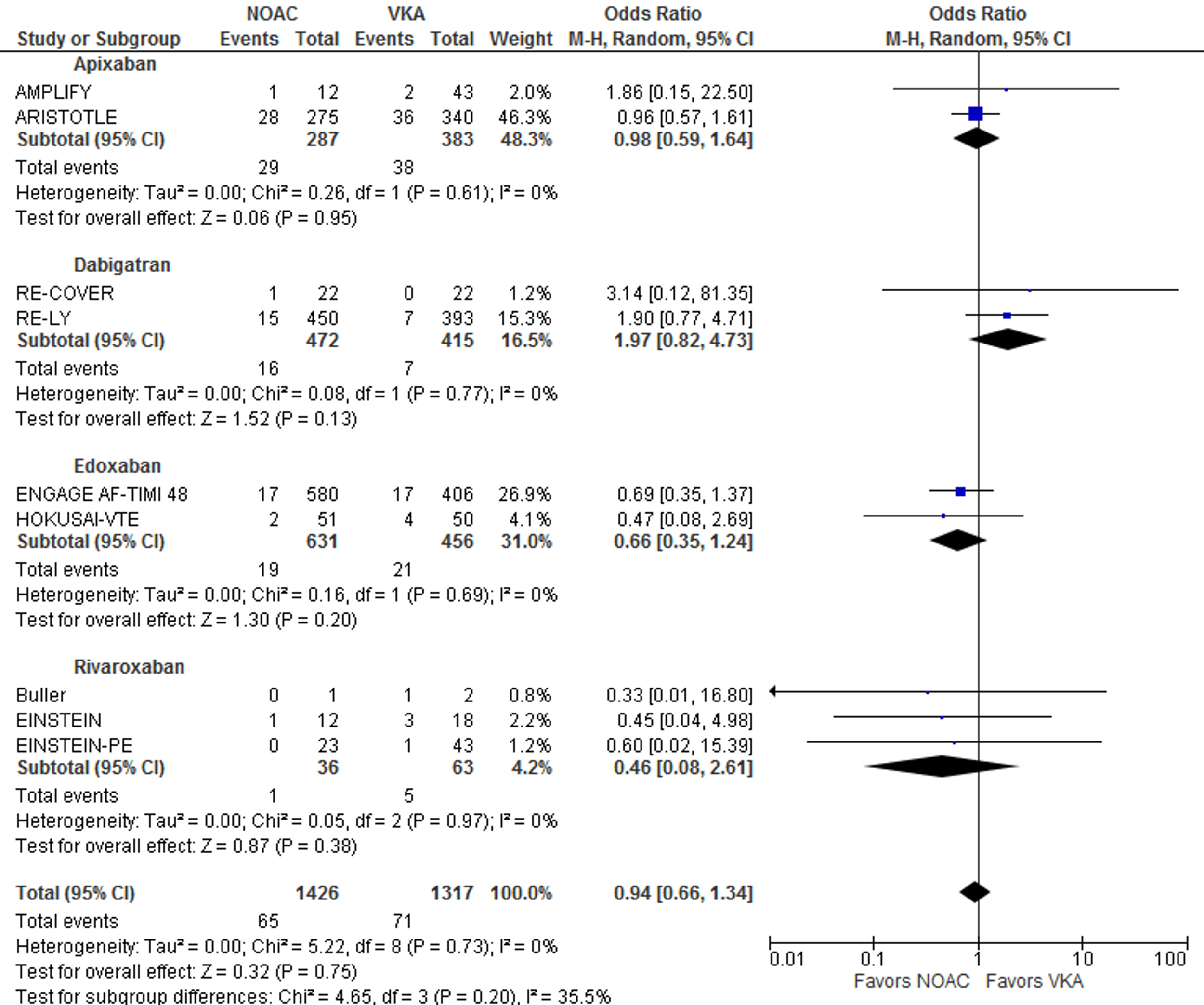

Supplement: S7 Fig — Odds ratios and 95% confidence intervals are given for the relative odds that major extracranial bleeding events will lead to fatal bleeding events. Calculations were made using the number of total bleeding events for the following studies: EINSTEIN, EINSTEIN-PE, ENGAGE AF-TIMI 48, HOKUSAI-VTE, J-ROCKET, RE-COVER, RE-COVER II, RE-LY AND RE-MEDY. (TIF) [file pone.0137444.s009.tif]
