# Supplementary material for: Risk of Fatal Bleeding in Episodes of Major Bleeding with New Oral Anticoagulants and Vitamin K Antagonists: A Systematic Review and Meta-Analysis
Source: PLoS One. 2015 Sep 18;10(9):e0137444. doi: 10.1371/journal.pone.0137444 (PMC4575170; doi:10.1371/journal.pone.0137444)
Supplement: S8 Fig — Example of the search strategy used to systematically review the studies indexed in the Cochrane database. (PDF) [file pone.0137444.s010.pdf]

Appendices

2 Cochrane search strategy

Search Name: NOAC meta PICOS

Date Run: 20/08/14 08:56:25.925
ID Search Hits

#1 MeSH descriptor: [Embolism and Thrombosis] explode all trees 5797

#2 MeSH descriptor: [Atrial Fibrillation] explode all trees 2677

#3 MeSH descriptor: [Stroke] explode all trees 5352

#4 MeSH descriptor: [Thromboembolism] explode all trees 1711

#5 MeSH descriptor: [Pulmonary Embolism] explode all trees 914

#6 MeSH descriptor: [Venous Thromboembolism] explode all trees 409

#7 {or #1-#6} 13065

#8 dabigatran:ti,ab,kw 226

#9 apixaban:ti,ab,kw 131

#10 rivaroxaban:ti,ab,kw 240

#11 edoxaban:ti,ab,kw 41#12 #7 and {or #8-#11} Publication Year from 2000 to 2014, in Trials 121
