# Supplementary material for: Risk of Fatal Bleeding in Episodes of Major Bleeding with New Oral Anticoagulants and Vitamin K Antagonists: A Systematic Review and Meta-Analysis
Source: PLoS One. 2015 Sep 18;10(9):e0137444. doi: 10.1371/journal.pone.0137444 (PMC4575170; doi:10.1371/journal.pone.0137444)
Supplement: S1 Table — Tool used to extract data from included studies. (PDF) [file pone.0137444.s012.pdf]

Appendices

1 S1 Data extraction sheet

Trial	Control (C)	C age	I major bleeds #	C fatal GI bleed #
Indication (VTE, AFIB)	PMID/NCT	I % age > 75	C major bleeds #	I major intracranial bleeds #
Exclusions	primary data=1; duplicate data=0	C % age > 75	I fatal bleeds #	C major intracranial bleeds #
Study type	Bleed definition (ISTH v. other)	I % GFR<50	C fatal bleeds #	I fatal intracranial bleeds #
Sequence generation (1=low risk, 0=unclear, -1=high risk)	I withdrawal %	C % GFR<50	I non-fatal major bleeds #	C intracranial bleeds #
Allocation concealment	C withdrawal %	I % previous CVA/TIA	C non-fatal major bleeds #	I extracranial major bleed #
Blinding	I study size	C % previous CVA/TIA	I major gastrointestinal bleeds #	C extracranial major bleed #
Incomplete outcome data	C study size	I % antiplatelet use	C major gastrointestinal bleeds #	
Intervention (I)	I age	C % antiplatelet use	I fatal GI bleed #	
