# Supplementary material for: Risk of Fatal Bleeding in Episodes of Major Bleeding with New Oral Anticoagulants and Vitamin K Antagonists: A Systematic Review and Meta-Analysis
Source: PLoS One. 2015 Sep 18;10(9):e0137444. doi: 10.1371/journal.pone.0137444 (PMC4575170; doi:10.1371/journal.pone.0137444)
Supplement: S2 Table — Values are percentages of patients in each treatment arm with the individual characteristics at baseline. Fisher’s exact test was used to calculate p-values. (PDF) [file pone.0137444.s013.pdf]

**Category**	**Rivaroxaban**	**Warfarin**	**P-value**
Age <65	14.9%	15.3%	0.921
Age 65-74	28.6%	31.9%	0.350
Age >=75	56.5%	52.8%	0.315
Male	65.8%	65.5%	1.000
Female	34.2%	35.0%	0.822
White	84.1%	78.0%	0.036
Black	1.5%	0.8%	0.506
Asian	11.1%	18.1%	0.006
Other	3.3%	3.1%	1.000
BMI <=25	22.3%	26.4%	0.183
BMI 25 to <=35	62.5%	61.7%	0.825
BMI >35	15.2%	11.9%	0.210
CrCl <50	25.1%	26.2%	0.743
CrCl 50 to 80	46.3%	50.8%	0.224
CrCl >80	28.4%	23.1%	0.102
CHADS 2	14.7%	12.7%	0.467
CHADS 3	43.3%	45.1%	0.666
CHADS 4	29.1%	28.2%	0.813
CHADS 5	10.9%	12.4%	0.506
CHADS 6	2.0%	1.6%	0.789
No prior stroke/	52.9%	51.8%	0.775
Prior stroke/TIA/	47.1%	48.2%	0.775
No congestive h	41.0%	39.6%	0.716
Congestive hea	59.0%	60.4%	0.716
No hypertensio	9.9%	9.6%	0.905
Hypertension	90.1%	90.4%	0.905
No diabetes	58.2%	56.2%	0.613
Diabetes	41.8%	43.8%	0.613
No prior MI	79.2%	81.9%	0.367
Prior MI	20.8%	18.1%	0.367
Persistent AF	81.8%	81.6%	1.000

Paroxysmal AF	16.7%	16.8%	1.000
New onset AF	1.5%	1.6%	1.000
North American	37.7%	28.8%	0.008
Latin American	11.6%	10.6%	0.733
Western Europe	12.4%	17.9%	0.036
Eastern Europe	22.3%	21.8%	0.863
Asia Pacific	15.9%	21.0%	0.079
No prior ASA us	56.7%	58.8%	0.563
Prior ASA use	43.3%	41.2%	0.563
No prior VKA us	31.6%	35.5%	0.257
Prior VKA use	68.4%	64.5%	0.257
